# Editorial: Mobile health interventions to address maternal health: ideas, concepts, and interventions

**DOI:** 10.3389/fdgth.2024.1378416

**Published:** 2024-02-29

**Authors:** Avishek Choudhury, Ashish Nimbarte

**Affiliations:** Industrial and Management Systems Engineering, West Virginia University, Morgantown, WV, United States

**Keywords:** maternal health, mobile health (mHealth), underserved and unserved populations, digital divide, health equity

**Editorial on the Research Topic** Mobile health interventions to address maternal health: ideas, concepts, and interventions

In the global endeavor to mitigate maternal mortality, particularly within disadvantaged populations, the potential of mobile health (mHealth) interventions is increasingly recognized as a pivotal element (1). This fact highlights a profound disparity in the accessibility and quality of healthcare services. mHealth technologies, leveraging the ubiquity of mobile phones, offer a promising pathway to bridge these gaps, presenting a novel approach to enhancing maternal health outcomes in underserved communities (2). Moreover, mHealth initiatives empower women by placing vital health information and resources at their fingertips. This empowerment supports informed decision-making and promotes autonomy in health-related matters (3). Additionally, from a systemic perspective, mHealth solutions offer a scalable and cost-efficient method to augment the capacity of overburdened health systems. This endeavor not only saves lives but also represents a significant stride toward bridging the healthcare divide, ensuring that every woman has the opportunity for a safe pregnancy and childbirth.

Despite the plethora of mHealth technologies available today, maternal health outcomes in underserved communities remain disproportionately poor (4, 5). This ongoing challenge can be largely attributed to the lack of an integrative approach in deploying these technologies. Much of the research and implementation efforts overlooks factors such as cultural nuances, user perceptions, ecological validity, and the sustainability of technology within specific community contexts (6). Such oversight leads to a disconnect between the intended benefits of mHealth solutions and their actual adoption and effectiveness on the ground. To bridge this gap, what is urgently needed is a user centered inclusive and integrative approach that respects and incorporate the unique cultural and societal dynamics of each community (3).

This editorial presents recent studies underscoring the potential of integrating mHealth solutions into maternal care, pointing towards a future where such interventions could significantly reduce disparities in maternal health outcomes. Through a lens of inclusivity, engagement, and cultural sensitivity, this editorial brings into focus the imperative for co-designed, user-friendly mHealth technologies that can cater to the diverse needs of communities worldwide, ensuring that every woman has access to the care and support she needs during one of the most critical times of their life. The convergence of research on mHealth solutions led by de Souza et al., Pierce et al., Rogers et al., and Donelle et al. offers hope and insights.

The collective insights from these studies underscore the imperative need for a nuanced, inclusive, and user-centered approach in the development and implementation of mobile health (mHealth) technologies, particularly in the domain of maternal health. The Research Topic highlights several key takeaways:

• **Technological inclusivity and accessibility:** The study by de Souza et al., from Brazil on remote chest feeding support illustrates the necessity for technologies that are integrative and user-friendly, capable of overcoming barriers to remote healthcare delivery in regions marked by socioeconomic disparities. The advocated hybrid model of in-person and remote consultations serves as a template for balancing effectiveness with accessibility, pointing to a broader principle of designing mHealth solutions that are inclusive and adaptable to diverse contexts.
• **Co-design with vulnerable populations:** The investigation by of the MyCare app by the Pierce et al. University College London Hospital team reveals the limitations of mHealth tools when they fail to reach vulnerable groups such as refugees, individuals facing mental health challenges, victims of domestic violence, and non-English speakers. This situation emphasizes the importance of co-designing digital tools with the input and involvement of the intended users, ensuring that these technologies do not inadvertently widen health inequalities but rather cater to the specific needs and circumstances of all user groups.
• **Alignment with social determinants of health:** Rogers et al.’s work in the United States demonstrates how mHealth solutions that are attuned to the Social Determinants of Health can effectively address systemic inequities. By incorporating insights from human factors and public health, mHealth interventions can be strategically designed to meet the needs of marginalized communities, offering a path to more equitable healthcare outcomes.
• **Digital health literacy:** Donelle et al.’s investigation in Canada on digital health literacy among new parents during the pandemic brings to light the double-edged sword of high digital engagement. While digital tools offer significant benefits, they also pose risks when not used judiciously. This finding stresses the importance of fostering balanced digital parenting practices and integrating digital health literacy into prenatal and postnatal education, ensuring that parents are equipped to navigate the digital health landscape effectively and safely.

Overall, these studies collectively argue for a shift towards mHealth solutions that are deeply rooted in the principles of equity, inclusivity, and user-centered design. Readers should take away the understanding that the success of mHealth interventions in improving maternal health outcomes and addressing broader healthcare challenges hinges on our ability to create technologies that are accessible, equitable, and responsive to the diverse needs of all community members.

Several vital areas emerge in addressing the future implications of these findings for policy and practice. First, the development of customized mHealth solutions is critical. These solutions should be crafted with an intimate understanding of the target population's specific circumstances, moving beyond a one-size-fits-all approach. Policymakers and healthcare providers must acknowledge the diversity of challenges across different communities and strive to meet these varied needs effectively. Another significant implication is the need to bridge the existing digital divide, ensuring that technological solutions are accessible and user-friendly for all groups, regardless of socioeconomic status, language proficiency, or cultural background. Integrating human factors and public health insights into mHealth design is paramount to ensure that mHealth solutions are mutually beneficial and widely adopted. Special attention to vulnerable populations is another critical area. Groups such as refugees, individuals dealing with mental health issues, and victims of domestic violence require particular focus (7, 8). Co-designing digital tools with such groups can ensure that their unique needs and challenges are adequately addressed, helping to reduce health disparities.

What's notably absent and should be the focus of future research is the development of strategies to foster intrinsic motivation among potential users of mHealth technologies. The true effectiveness of mHealth interventions will be realized when users remain engaged with these solutions beyond the lifespan of initial research projects. A prevalent challenge in specific societies and underserved communities is the existence of deeply rooted beliefs that potentially harm health outcomes, such as the notion that consuming less food during pregnancy will result in a smaller baby size, ostensibly simplifying childbirth (9–11). Future studies should explore how mHealth technologies can be leveraged to educate and gradually shift these populations away from harmful beliefs towards more healthful practices. This involves not just disseminating information but engaging users in a way that sparks their curiosity, fosters a sense of personal relevance, and empowers them to make informed health decisions autonomously. The aim would be to not only provide access to healthcare information but to also cultivate a mindset change those values scientific understanding and the importance of health, thus breaking the cycle of misinformation and improving health outcomes through sustained behavioral change.

In conclusion, the future of mHealth in maternal and parental support looks promising, but achieving its full potential requires a collaborative effort from governments, healthcare organizations, technology developers, and the communities they serve. The goal is to forge a mHealth ecosystem that is inclusive, responsive, and adaptable, catering to the needs of all users, especially those in marginalized communities. By tackling these challenges and harnessing the opportunities presented by digital technology, we can move towards a future where equitable, accessible, and effective digital healthcare is a reality for everyone.
